# Editorial: Soft Robotics Based on Electroactive Polymers

**DOI:** 10.3389/frobt.2021.676406

**Published:** 2021-04-30

**Authors:** Guoying Gu, Herbert Shea, Stefan Seelecke, Gursel Alici, Gianluca Rizzello

**Affiliations:** ^1^ Robotics Institute, School of Mechanical Engineering, Shanghai Jiao Tong University, Shanghai, China; ^2^ State Key Laboratory of Mechanical System and Vibration, Shanghai Jiao Tong University, Shanghai, China; ^3^ Soft Transducers Laboratory (LMTS), School of Engineering, École Polytechnique Fédérale de Lausanne (EPFL), Neuchâtel, Switzerland; ^4^ Department of Systems Engineering, Department of Materials Science and Engineering, Saarland University, Saarbrucken, Germany; ^5^ School of Mechanical, Materials, Mechatronic and Biomedical Engineering, University of Wollongong, Wollongong, NSW, Australia; ^6^ Applied Mechatronics and Biomedical Engineering Research (AMBER) Group, University of Wollongong, Wollongong, NSW, Australia

**Keywords:** electroactive polymers, soft robots, soft actuators, soft sensors, dielectric elastomer actuator

Conventional robots using rigid structural elements and high-output actuators have enabled great progress in automated assembly and manufacturing (Nof, 1999; Yang et al., 2018). An increasing number of robots need to interact safely with humans, manipulate soft and delicate objects or need to move in difficult environments. Soft robotics is a rapidly growing field as compliant robots are capable of sustaining large deformation while maintaining structural compliance, allowing safe operation with people and adaptation to many situations (Rus and Tolley, 2015).

Most soft robots rely on pneumatic actuation which requires a compressor or pump (Shintake et al., 2018). Electroactive polymers (EAPs) are smart soft materials capable of changing sizes and/or shapes in direct response to electrical stimuli (Bar-Cohen, 2002; Gu et al., 2017). They allow combining actuator, sensor and structure in one single topology. For these reasons, soft robotic systems with EAPs (ionic EAPs and Dielectric Elastomer Actuators) have gained great attention.

The goal of this research topic is to provide an overview of the state of the art in design, fabrication, modeling, control and applications of soft robotic systems based on both EAPs. After the peer review process, this topic accepted 13 articles, including 11 research articles and 2 review articles.

There are four articles focusing on the actuator, sensor and structure design. Cacucciolo and co-authors (Cacucciolo et al) describe a new class of electrically driven soft fluidic muscles combining thin McKibben actuators with stretchable pumps based on ElectroHydroDynamics, to directly pump liquid molecules by means of an electric field. This architecture does not require an external pump or compressor, easing integration in textile and wearables. Hashimoto et al. present a novel 3D self-folding structure by combining a rigid frame with a stretchable dielectric elastomer actuator. The novel design has a simple structure, allowing for high-speed and high-precision operations. The proposed design is employed to develop a three-joint gripper which can grasp an object with length of 36.7 mm and weight of 3.28 g. Must et al. explore the use of soft and compliant actuator material based on ionic and capacitive laminate (ICL) for potential use in miniaturized manipulators. Three techniques are demonstrated, rendering a bulk of ICL into a practical manipulator, which is highly compatible with electron optics. After presenting experimental characterization results, the ability of the ICL manipulator in handling a single cell is experimentally demonstrated. Shintake et al. report soft stretchable capacitive strain sensors whose gauge factor is doubled from 0.8 to over 1.6 by using auxetic structures. The sensors consist of liquid metal electrodes separated by an elastomer film, on which silicone auxetic structures exhibiting negative Poisson ratio have been bonded. The authors measure the change in capacitance as the sensor are stretched, measuring capacitance vs. strain. The sensors are robust, with no change in sensitivity after 11,000 cycles under 50% strain.

There are four articles focusing on the robot design and application. Franke et al. present a dielectric elastomer actuator (DEA) driven soft robot consisting of two DEA artificial muscles on the top and bottom of a soft silicone body. This design prevents the undesired bending when a pre-stretched DEA membrane is applied on the soft robotic structures using a bioinspired stiff skeleton. The robotic structure shows a large and defined bimorph bending curvature under both static and dynamic actuations. An analytical model is developed to study the effects of applied voltage, material stiffness and pre-stretch ratio. A prototype fabricated after the optimal design shows that fast and large dynamic displacements of about 47 mm can be accomplished, while blocking force can be optimized from 1 to 4 mN. Pfeil et al. present a bioinspired worm-like crawling soft robot based on dielectric elastomer actuators, which are cylindrical actuator segments used to set the structure in motion. Textile materials consisting of parallel oriented carbon fibers are used to enhance the stiffness in the supporting areas of the robot. Christianson et al. demonstrate the swimming performance of an untethered jellyfish-inspired robot, propelled by dielectric elastomer actuators. An axisymmetric array of unimorph actuators based on non-prestretched DEAs are used to provide upwards propulsion. After experimentally characterizing and modeling the actuators, they have designed a waterproof power supply to drive the soft actuators based on DEAs. The robot swims at an average speed of 3.2 mm/s. Olsen and Kim present a design method for a small aquatic robot's swimming mechanism based on the jet propulsion mechanism found in jellyfish. Two modeling approaches are proposed: 1) a geometric model to gain qualitative information about the feasibility for the robot design, and 2) a refined mechanics model based on a linear beam theory and an equivalent circuit model. Both models can be used to improve the performance of the P1 swimming mode as compared to that of a typical jellyfish.

There are three articles focusing on the modeling and control. Nalbach et al. report on modeling and design optimization of multi-degrees of freedom (MDOF) dielectric elastomer actuator (DEA) for soft robotic applications. The actuator consists of a pair of cone DEA membranes whose electrodes are partitioned in four sections to be activated separately in order to generate the desired MDOF actuation feature. After experimental characterization, a mathematical model is developed to design optimize a prototype capable of performing bending angles of about 9.6 degrees. Chi et al. develop an iterative learning control method to track the motion trajectory of a circular soft crawling robot, which uses DEA as the robot body and four electro-adhesion actuators as feet. They use a knowledge-based modeling framework to establish the physical model of the DEA, and employ ILC for motion trajectory tracking control. Simulations and experimental results are presented to demonstrate the effectiveness of the developed soft robot and the control scheme. Hoffstadt and Mass report on closed-loop control of DE stack-actuators using a new self-sensing scheme that does not require a superimposed excitation signal. The self-sensing system is experimentally validated, showing performance at up to 400 Hz equivalent to what can be obtained using external sensors. The control of voltage, DEA force or DEA displacement can be achieved by using different feed-forward control structures.

There are two review articles. Hao et al. reviews the current trends, challenges and potential solutions of IPMC as soft actuators and sensors. This review investigates the key factors influencing the actuating performance of IPMC, and potential of IPMCs as soft sensors. Hu et al. provide a review of state-of-the-art PEDOT-based conducting polymer actuators. This review also provides a comprehensive understanding of the actuation mechanism, performance evaluation criteria, fabrication technologies, and possible applications of PEDOT-based actuators. It discusses two actuation mechanisms and three actuation configurations. Evaluation criteria for actuator performances and material properties are surveyed, and the effect of electrode and electrolyte materials are summarized. After presenting their applications in the bionics and biomedicine area, textiles and wearable devices and micro applications, four future research opportunities to improve and widen the applications of PEDOT-based actuators are discussed.

We hope that the articles presented in this research topic can provide in-depth insights and understandings in the field of soft robotics involving with the electroactive polymers and inspire new design of bio-inspired and robust soft robots for comprehensive applications.
